# Newly Qualified Teachers’ Well-Being During the COVID-19 Pandemic: Testing a Social Support Intervention Through Design-Based Research

**DOI:** 10.3389/fpsyg.2022.873797

**Published:** 2022-06-07

**Authors:** Dominik E. Froehlich, Julia Morinaj, Dorothea Guias, Ulrich Hobusch

**Affiliations:** ^1^Centre for Teacher Education and Department of Education, University of Vienna, Vienna, Austria; ^2^Department of Research in School and Instruction, Institute of Educational Science, University of Bern, Bern, Switzerland; ^3^Department of Education, University of Vienna, Vienna, Austria; ^4^Centre for Teacher Education, University of Vienna, Vienna, Austria

**Keywords:** design-based research, newly qualified teachers, social support, well-being, teachers

## Abstract

Around the world, newly qualified teachers are leaving the profession after only a short time working at school. This not only has a negative effect on the capacities of the respective education systems, but also for the teachers themselves, as it often due to factors such as stress and burnout that leads to this decision. The COVID-19 pandemic has exacerbated this situation by adding to the teachers’ workload, uncertainty, and stress. Previous research has investigated strategies that may help teachers improve their well-being and, among other factors, found social support to be an important condition. In this mixed methods design-based research study, we developed a design to enhance social support among newly qualified teachers in their first months working at school. Our quantitative and qualitative results show that the design has positive effects on many aspects of teachers’ well-being in the intervention group both longitudinally (before and after the intervention) and when compared to a comparison group. The findings are being discussed considering the recent changes in the working conditions of teachers as imposed by the COVID-19 pandemic.

## Introduction

It is a global phenomenon that newly qualified teachers (NQTs) often leave the teaching profession within the first few years after graduation from a preservice educational program (Fantilli and McDougall, 2009; Hentges, 2012; Smith and Ulvik, 2017; Weldon, 2018). But the COVID-19 pandemic has even exacerbated the situation (Pressley et al., 2021), as it brought a whole new set of demands for teachers. Many countries experienced a total lockdown involving the immediate closure of schools and universities (Flores and Swennen, 2020) and educators at schools and teacher education institutions had to swiftly adapt to remote teaching and learning to carry on their job duties (Quezada et al., 2020). Unexpected situations such as COVID-19 aggravated the challenges with which (novice) teachers are confronted under normal circumstances; studies report increased stress and a higher propensity for depression and burnout (Anderson et al., 2021; Pressley, 2021; Santamaría et al., 2021). Importantly, these new demands also need to be seen in the context of generally increased stress levels due to the pandemic itself—for example, through health issues in family or oneself, or caretaking responsibilities (Froehlich, 2021)—and the probability of increasing them even more (Bakker et al., 2005; Bakker and Demerouti, 2007). Also in the Austrian context, a recent study showed that the additional workload and the lack of digital skills are a great burden for teachers (Woltran et al., 2021).

Intention to leave the profession is associated with various factors, including a lack of helpful collegial relationships in preservice education, low-quality orientation programs, unhelpful mentoring, dissatisfaction with working conditions (e.g., high workload), disappointment with the school system, and a lack of commitment (Kelly et al., 2019; Räsänen et al., 2020). On the other hand, turnover intention has been found to decrease when teachers experience a higher degree of well-being (Arnup and Bowles, 2016; Chang et al., 2017; Grant et al., 2019; White and McCallum, 2020). Hence, teachers’ well-being can serve as an important catalyst for commitment to the teaching profession and intentions to remain in their current job (Klusmann et al., 2008; McCallum, 2020). It was repeatedly found that teacher social and emotional well-being are strongly related to the teaching quality and that it is instrumental in enhancing pupils’ success, achievement, and satisfaction (Jennings and Greenberg, 2009; White and McCallum, 2020). One key to overcoming turnover intention may be found in social support. Social support, the “perception or experience that one is cared for, esteemed, and part of a mutually supportive social network” (Taylor, 2011, p. 192), has been found to increase well-being (Helliwell et al., 2014; Beausaert et al., 2021) and the competencies necessary to navigate the current volatile context (Froehlich, 2021; Van Tilburg et al., 2021).

But while some basic research on the topic exists, this did not yet help to solve the problem in practice so far. Therefore, we attempt to build a bridge to translate the existing theories into a specific implementation in practice. This is even more relevant, since this is about an implementation during the COVID-19 pandemic—and we yet do not fully understand how the increased use of digital means affects the concept of social support (Froehlich, 2021). To provide a cost-efficient solution, we are interested in one specific form of social support, namely, the social support received from peers (i.e., from other NQTs), especially through digital exchange (due to the constraints imposed by COVID-19). Specifically, we seek to explore how NQTs’ well-being may be enhanced through social support in a digital setting: Does a digital intervention to increase peer social support help NQTs to develop greater well-being? For this, we apply design-based research (A. Bakker, 2018), a scientific approach of developing practical interventions (“designs”) and testing them in the field with the goals of (a) learning something new for science and (b) having a direction impact in the field. In particular, we devise a theory-driven design (Bakker and Van Eerde, 2015) to foster social support in a digital setting and teachers’ well-being. This design is then evaluated using qualitative and quantitative data.

Through this intervention and subsequent evaluation, we aim to make the following two major contributions. First, we study digital social support in the volatile context of COVID-19 and how it may help with improving NQT’s well-being. While there has been some focus in the literature on NQT’s social support in recent years (e.g., Cooper and Stewart, 2009; Baker-Doyle, 2012; Moolenaar, 2012; Thomas et al., 2020), we extend this research by focusing on social support delivered through digital means and NQT’s in the context of a global pandemic. It is indeed important to re-check the usefulness of social support in this digitized context, as the forced reliance on more digital means of communication may have changed the nature of social support quite fundamentally and, therefore, more research in this direction has been called for (Froehlich, 2021). Second, the approach of design-based research is also very fruitful in terms of the practical implications we can derive from it. The design is not only an important element of this research to generate new knowledge, but also a template to provide easily applicable strategies aimed at increasing teachers’ social support and, in turn, well-being. Again, this is much needed especially during the crisis condition, in which the public funds are severely constrained by higher public health expenses (*cf*. Coccia, 2021). We aim to make these contributions in three steps: In the first part, we review the theoretical approaches that we used to develop the design. We then describe how we implemented the design and collected and analyzed data both quantitatively and qualitatively. For the quantitative analyses, we checked for differences of the intervention group with a comparison group (Hypothesis 1), tested for differences across measurement points of the intervention group (Hypothesis 2), and performed an analysis about the perception of the social processes within the peer groups formed in our intervention (Hypothesis 3). With the qualitative analyses, we inductively investigated the most prevalent and important themes regarding the NQTs’ well-being, their perception of the peer support, as well as their reflection on the peer support group. Last, we present and discuss the results and derive implications for both research and practice.

## Background

### Teacher Well-Being

Despite the importance of teacher well-being, there is little consensus on its definition. Well-being in the teaching profession is primarily associated with job satisfaction and is often described in deficit terms: for example, a lack of stress, burnout, emotional exhaustion, or problems with retention (Roffey, 2012; Schiefele et al., 2013; Hall-Kenyon et al., 2014; Mattern and Bauer, 2014; Breeman et al., 2015; Yildirim, 2015; Lavy and Eshet, 2018). Other researchers defined teacher well-being as a positive emotional state, resulting from harmony between teachers’ environmental and personal factors (e.g., Aelterman et al., 2007; Brouskeli et al., 2018) and as a main driver of teacher effectiveness (Duckworth et al., 2009). A recent systematic review of the research literature on teacher well-being (Hascher and Waber, 2021) showed that the definition and the operationalization of teacher well-being differ across the studies. Studies on teacher well-being vary in regard to the number and choice of subdimensions, whereby the predictors, indicators, and outcomes of teacher well-being are not clearly differentiated. Hascher and Waber (2021) provided support for the multidimensional approach to teacher well-being, in which positive dimensions outperform negative dimensions and suggested to consider the specific working context of the teaching profession. Many scholars agree that well-being is best understood as a multidimensional concept, consisting of several distinct but related dimensions (e.g., Fraillon, 2004; Soutter et al., 2014; Borgonovi and Pál, 2016). For example, building on Ryff (1989) and Warr’s (1994) theoretical conceptualizations, Van Horn et al. (2004) proposed a multidimensional model for occupational well-being, consisting of affective, cognitive, professional, social, and psychosomatic dimensions. Although the model was designed for the specific working context of teachers, there was no clear definition of (teacher) well-being, which does not allow to understand the reasons for the selection of the five dimensions. As with any multidimensional model, it may be necessary to investigate the relationships between the dimensions of well-being within the model. Hascher (2004, 2012) introduced a multidimensional model of well-being in the school context, comprising positive and negative dimensions, which can be used as indicator categories of well-being in school. The model has primarily been used in the context of student well-being and address the subdimensions address cognitive, affective, and physical elements. According to Hascher (2004), well-being in school can be conceptualized as the prevalence of positive emotions and cognitions toward school, persons in school, and the school context over the negative feelings and cognitions toward school life. A high degree of well-being in school indicates the dominance of positive experiences (i.e., positive attitudes toward school, enjoyment in school, and positive academic self-concept) over the negative ones (i.e., worries in school, physical complaints, and social problems in school).

The focus on and applicability in the school setting allowed to introduce the model regarding well-being in the teaching profession. Accordingly, teachers would have higher levels of well-being when they experience more positive emotions and cognitions versus less negative ones. Teacher well-being may thus be viewed as “a positive imbalance,” with the prevalence of positive aspects (Hascher and Waber, 2021). Teachers may simultaneously experience feelings of joy, happiness, and satisfaction in teaching along with worries, stress, frustration, and physical complaints associated with interactions with students or their parents, or pressures in the work environment such as lack of time and work overload (Soini et al., 2010). Positive experiences in school can coexist with negative experiences like positive dimensions of well-being can coexist with negative ones (Collie and Martin, 2017; Lavy and Eshet, 2018). Investigating positive aspects such as positive emotions at work simultaneously with the negative ones such as worries or physical complaints was strongly suggested by the recent systematic review on teacher well-being (Hascher and Waber, 2021), because the predominance of positive experiences does not exclude the existence of the negative ones. Teachers’ meaningful interactions with students and colleagues or a deeper sense of teaching may function as buffers against the job demands and challenging situations teachers face in school. Similarly, if teaching is evaluated as successful and in line with its objectives, this leads to satisfaction and teacher well-being (Bieri, 2006).

In this study, we follow the multidimensional model of well-being in school (Hascher, 2004), which allows us to simultaneously scrutinize teachers’ positive as well as negative experiences in the school context and encompasses factors that are specifically related to the teaching profession.

### Social Support

The construct of social support is defined and operationalized differently across studies. For example, Cobb (1976) has argued that social support is “information from others that one is loved and cared for, esteemed and valued, and part of a network of communication” (p. 300). More recent research is increasingly differentiating between the various forms and types of social support (Rhodes, 2004; Froehlich et al., 2017). Hogan et al. (2002) reviewed 100 studies and illustrated that social support can be defined in more general terms as “an exchange between providers and recipients” and described three major types of supportive social interactions (Hogan et al., 2002, p. 382). First, emotional support entails care and concern which can be expressed through verbal and nonverbal communication. Second, informational support involves the provision of information which can be useful for a recipient. Third, instrumental support pertains to the provision of services and material commodities. Similarly, a study involving schoolteachers measured perceived social support using instrumental (provision of assistance and services) and emotional support (provision of caring behaviors and understanding; Ju et al., 2015).

In addition, social support can come from a variety of sources (e.g., family, friends, peers, and supervisors; Ford et al., 2007). French et al. (2018) differentiated between support forms (behaviors vs. perceptions), sources (broad vs. specific), and types (instrumental vs. emotional) and defined social support as “psychological or material resources provided through social relationships that can mitigate strains” (p. 288).

Previous research has repeatedly shown that social support plays an essential role in well-being (Chu et al., 2010; Rueger et al., 2016; Van Tilburg et al., 2021) and interacts with coping following stressful experiences (Bal et al., 2003). Positive and supportive relationships with significant others may serve as a coping resource in teachers’ lives when faced with a stressful or challenging event in school. Building on Lazarus and Folkman’s (1984) appraisal model of stress, assuming that stress results from imbalances in demands and resources, McCarthy et al. (2016) suggested a model of the appraisal process with teachers. According to this model, teachers appraising their social support (as resources) as equal to or exceeding demands they face in the teaching profession will experience less stress and feel more satisfied with their job, which may result in teacher well-being. On the contrary, teachers appraising their social support as insufficient will be more likely to experience stress, dissatisfaction with their job, and low levels of well-being. This model can therefore be used to explain why some teachers keep going while others decide to leave the profession. To put it differently, teachers are likely to benefit from social support and express more favorable well-being when they believe that they have supportive social networks and know that someone can help when they are in need.

Especially during the first few years in the teaching profession, the availability and quality of social support as well as socialization structures (e.g., teacher induction and mentorship support) can play a crucial role among NQTs in their decisions to stay in or leave the profession (Joiner and Edwards, 2008; Fantilli and McDougall, 2009). Many studies confirmed that social support can serve as an important resource to cope with work overload, stress, and burnout (Maslach et al., 2001; Brackett and Mayer, 2003; Beausaert et al., 2016, 2021; Harwood and Froehlich, 2017). Involvement in social support groups of peers can provide NQTs an opportunity to develop meaningful social networks and resources to cope with stressful circumstances and job demands. The peer support group intervention of Maton (1988) suggested that it has beneficial effects on well-being and group satisfaction. This intervention also showed that bidirectional supporters reported higher well-being compared to those who engaged in only providing or receiving support.

To summarize, perceived social support and active involvement in social peer support groups may serve as a catalyst promoting NQT’s well-being. The existing literature on the relationship between social support and well-being served as a basis for the intervention study with the primarily goal to investigate whether NQTs’ well-being can be enhanced through social support.

## Materials and Methods

As stated above, we seek to explore how NQTs’ well-being may be enhanced through social support in a digital setting. We do so by focusing on one specific design of a digital peer support group. We approach our research question through the lens of design-based research (DBR; Bakker and Van Eerde, 2015; Bakker, 2018). DBR describes a research approach increasingly used by education researchers. As a fundamental idea, DBR involves developing interventions (“designs”) for real-world problems. Once a problem (field) is identified, customized interventions are then developed by referencing theory. Then, these interventions are deployed in practice and thoroughly evaluated. In essence, this approach not only tries to explore scientifically relevant knowledge (e.g., here we seek to offer another lens on how NQT’s well-being may be improved), but also contains a dimension of action (as in action research): the well-being of the participating NQTs should be improved directly; a useful tool is being developed also for subsequent cohorts of NQTs (Froehlich et al., 2021). In this article, we mix quantitative and qualitative data and analyses so that these two strands of research complement each other in evaluating the design (Schoonenboom et al., 2018). Specifically, the quantitative analyses contribute information about whether the sample is somehow different or biased from the outset (see Hypothesis 1), how well-being of the NQT developed longitudinally during the intervention (see Hypotheses 2), and how the NQT’s rate this process subjectively. The qualitative analyses contribute a list of topics that the NQTs were thinking about during the intervention that reflected the support they received from the peer group and its impact on their well-being. Also, they shared direct opinions about the intervention. The quantitative and the qualitative strand are integrated when discussing the results. In the following, we explicate the participants and design and then present how we collected and analyzed evaluative data to test the design.

### Participants

The study participants (*n* = 74 of which 42 are in the intervention group) were NQTs at Austrian secondary schools in 2021 (i.e., during the COVID-19 pandemic). All of them, both members of intervention group and the comparison group, who were attending the same course about self-reflection and evaluation of their own teaching practice as part of their further education; when signing up, they had no knowledge of the study. In other words, group allocation happened in a quasi-random manner. Members of the intervention group were informed about the study and data collection, the reasons behind the study, as well as all relevant procedures. Participation required consent, was fully voluntary and not part of the course in any way (filling in the surveys was additional workload, it was not necessary to participate in the study, no bonus points were given, etc.).

This means that all of them were pursuing a Master’s degree in teacher education, whereby entry into the school system usually happened shortly (estimate: usually 0–1 years) before that with the completion of the preceding Bachelor’s program in teacher education. Importantly, the study participants were not colleagues at the same school (as sharing a formal context may bias these relationships over others in the group; Meredith et al., 2017). Also, the sample is not restricted to certain subjects. The course was designed to encourage reflection on various topics related to everyday school life and to support NQTs in developing strategies for evaluating their own teaching practice.

Since this study was conducted during the COVID-19 pandemic, it is also important to describe the wider context of what it meant to be a teacher or NQT at this point in time. While the standard mode of teaching was in the classroom, the teachers and NQTs needed to adapt quickly to digital, hybrid, or blended teaching as thousands of classrooms and hundreds of schools were quarantined and closed throughout the semester.

### Design

For the intervention group, a new design was introduced, in which the NQTs were randomly assigned to peer social support groups. The group meetings were held over the course of one semester and included four to five meetings with pre- and post-meetings guided by the workbook. To give the NQTs the maximum amount of time for exchange and building social relationships, the group size was limited to four participants.

To provide an adequate and meaningful framework for the NQTs, the support groups were accompanied by a workbook, which suggested a structure for meetings and the overall make-up of the design. Before each of the peer support group meetings, which latest approximately 40 min, the *Meeting Planning* section asked the individual participants to identify topics that are relevant to them and that could be discussed in the group meeting. This section included guiding questions such as “What is my main topic or goal for this week?,” “Why is this topic important to me?,” or “What can I do to get more perspective about this topic?.” The *Meeting Session* section, as core part of the peer support group meeting, was structured in three stages, starting with a quick *check-in* phase (10 min), the *hot seat* (20 min), and the final *commitment* phase (10 min). The *check-in* phase provided data on the most important issues raised by participants within the previous week. The most pressing current issues and challenges of each participant were then addressed in the *hot seat* phase. Through the questions of the other group members, this phase may lead to new aspects and thus open new self-reflective perspectives. The final *commitment* phase was designed to lead participants to formulate a concrete plan for the upcoming week either to adopt changed perspectives on challenges, test new interventions, or address new issues. Participants were also given a space to record key points resulting during the group meeting. The *Meeting Review* section was designed to help participants reflect on any issues that have arisen during a meeting, approaches to solutions, or changing perspectives. We also designed a space for providing critique regarding the social support group meetings themselves to keep track of the meetings and, if necessary, improve them. Guiding reflective questions for this section include as: “What worked well in terms of the particular topic?,” “What could I improve?,” or “What could we change with regard to the group to make it run even better?”

### Instruments

To determine an effect of social support, NQTs in the intervention group completed a questionnaire on *teacher well-being* and *social support* after each of the five group meetings. In addition, a pre-test was administered at the beginning of the study before the first intervention (peer group meeting); a post-test was administered after all (five) peer group meetings. In sum, there were seven measurement points from t0 to t6. Details on the structure and items of the teacher well-being and social support questionnaires are presented in the next section.

In addition to the quantitative survey, the workbook immanent individual reflections, preparations, and post-processing provided an opportunity to evaluate complementary content and process dimensions of the meetings. Put differently, while the quantitative data allow for making comparisons across measurement points and in relation to a comparison group, the qualitative data permit also more open analyses that investigate whether social support processes or dimensions of well-being have been addressed outside of the quantitative measurement instruments, which all have been validated before COVID-19.

*Teacher well-being* was assessed with the 23-item *Teacher Well-being Questionnaire* (Hascher, 2020), including six distinct dimensions of well-being: (1) positive attitudes toward school (4 items; e.g., “I like to work in school,” *α* = 0.79), (2) enjoyment in school (4 items; e.g., “Have you experienced joy in the past few weeks because your lessons went well?,” *α* = 0.90), (3) positive academic self-concept (3 items; e.g., “I do not have problems coping with the demands at school,” *α* = 0.78), (4) worries in school (3 items; e.g., “Have you been worried in the past few weeks about school?,” *α* = 0.90), (5) physical complaints in school (5 items; e.g., “Have you had in the past few weeks a severe headache because of school?,” *α* = 0.87), and (6) social problems in school (4 items; e.g., “Have you had in the past few weeks problems with your colleagues?,” *α* = 0.75). NQTs responded to statements on a 6-point Likert scale (1 = *never/disagree*; 5 = *very often*/*agree*). The scale was tested in a recent pilot study with a sample of Swiss secondary school teachers and the reliability was Cronbach’s *α* = 0.68–0.87.

The perception of *social support* was measured with the 7-item *Situation in the Team* scale at time points t1 through t6 (Diel and Schmitt, 2010; e.g., “In our support group there is a good social climate” or “In our support group we trust each other”; the word “team” from the original scale has been replaced with “support group” to fit our context). Responses were indicted on a 5-point Likert scale ranging from 1 = *strongly disagree* to 5 = *strongly agree*. The internal reliability of the scale was excellent (*α* = 0.99). Additionally, we used Skaalvik and Skaalvik’s (2011) three items measuring *relations with colleagues* adapted for the peer group context (e.g., “Teachers in this peer group help and support each other,” *α* = 0.90). The scale was tested in a pilot study with Swiss secondary school teachers and the reliability was Cronbach’s *α* = 0.92.

The data from the quantitative surveys are completed by qualitative answers and reflections on prompts in the workbook (see description of the Design section above).

**Figure 1:**
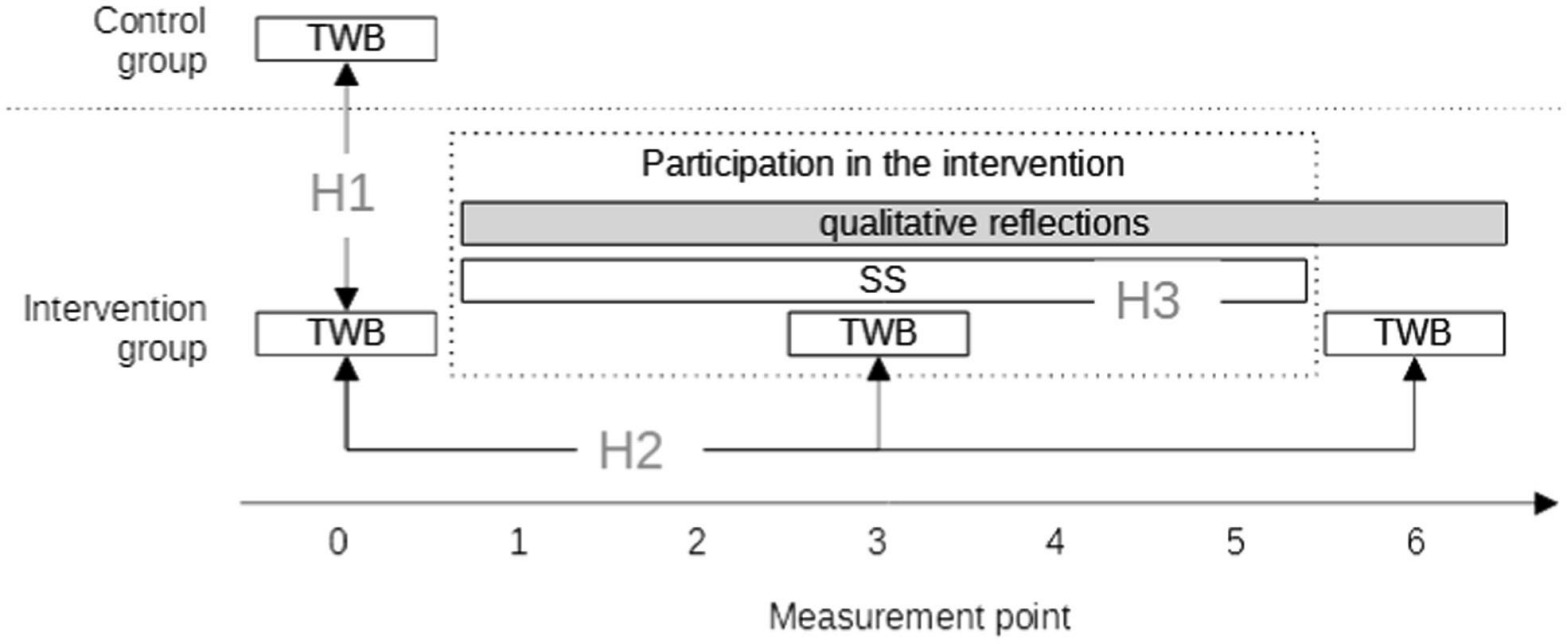
Temporal setup of the study. TWB, Teacher Well-Being; SS, Social Support, and H, Hypothesis.

Figure 1 outlines the temporal setup of the study.

### Quantitative Analyses

First, to check the assumption that the intervention group matches the comparison group in terms of well-being, we performed a cross-sectional check against NQTs enrolled in parallel courses using the Wilcoxon rank sum test (Hypothesis 1). Second, quantitative analyses also include the testing for differences across the measurement points of the intervention group (Hypothesis 2). As the data were mostly non-normal, we applied the Friedman test across three time points (t0 = pre-test, t3 = midpoint, and t6 = post-test). *Post hoc*-tests were corrected by the Bonferroni method. Last, we performed an analysis about the perception of the social processes within the peer groups (Hypothesis 3). Specifically, we evaluated the cross-sectional, subjective ratings of the questions about the functioning of the peer groups and the resulting relationships.

### Qualitative Analyses

We conducted qualitative content analysis (Mayring, 2021) on the corpus of text produced in the workbooks. Out of all workbooks for which consent was obtained (over 50), we analyzed 33 before the category system became saturated. We applied an inductive approach, which included the analysis of the open-ended questions in the workbook. Through this coding procedure we aimed to answer three questions: (1) What do the NQTs say about well-being and aspects related to well-being as discussed in literature? (2) What do the NQTs say about peer support? (3) What do the NQTs disclose about the peer support group? This process was carried out with QCAmap (Fenzl and Mayring, 2017). The qualitative analyses were led by the third author, but the coding procedures were regularly reviewed by and discussed with the first and second authors to safeguard accuracy and consistency. After the analyses were completed, the fourth author randomly sampled five workbooks from the full corpus (including those that were not analyzed due to theoretical saturation). He found that indeed all utterances of the NQTs can be mapped unto the existing category system and no revisions were suggested or made.

## Results

### Quantitative Results

For the quantitative analyses, we started with testing the assumption that the intervention group was indeed similar to the comparison group. For this test, we compared the data of the initial measurement of the intervention group with a comparison group sampled from parallel courses. We indeed did not find any differences for positive attitudes toward school (W = 211, *p* = 0.65), enjoyment in school (W = 435, *p* = 0.51), positive academic self-concept (W = 233, *p* = 0.97), worries in school (W = 463, *p* = 0.58), physical complaints in school (W = 555, *p* = 0.33), and social problems in school (W = 470, *p* = 0.85). These findings support Hypothesis 1.

In the next step, we compared the data of the intervention group across three measurement points using Friedman’s chi-squared (*χ*^2^) test. We found statistically significant differences for enjoyment in school [*χ*^2^(2) = 7.88, *p* < 0.05, statistically significant increases from t0 to t6], positive academic self-concept [*χ*^2^(2) = 21.11, *p* < 0.01, statistically significant increases from t0 to t6], worries in school [*χ*^2^(2) = 17.60, *p* < 0.01, statistically significant decreases from t0 to t6], physical complaints in school [*χ*^2^(2) = 8.64, *p* < 0.05, statistically significant decreases from t0 to t6], and social problems in school [*χ*^2^(2) = 11.08, *p* < 0.01, statistically significant decreases from t0 to t6]. While all these changes were as expected, statistically significant differences were not found for positive attitudes toward school [*χ*^2^(2) = 2.80, *p* = 0.25]. The data back up Hypothesis 2.

These findings are further supported by the data, as participants rated the social processes within the peer group to be functioning very well (*M* = 4.75 on a 5-point scale, *SD* = 0.74). They also expressed their content with relationship outcomes (*M* = 4.61 on a 5-point scale, *SD* = 0.70). The answers to both scales deviate significantly from a neutral opinion (the midpoint of the answer scale) and thus suggest to support Hypothesis 3 (based on the assumption that a very general population would answer in a balanced way, as we would expect for a thoroughly tested measurement instrument).

### Qualitative Results

Through the qualitative content analysis on the workbooks, we were able to distill the most prevalent and important themes regarding the NQTs’ well-being, their perception of the peer support given over the course, as well as their reflection on the peer support group. In the following, we present the results for these three main categories.

#### Well-Being

Well-being was discussed in all workbooks in various forms and regarding its different aspects and implications. Every NQT was preoccupied with at least one of these aspects. The participants showed awareness of the importance of work-life balance for their well-being considering the challenges and problems they were confronted with in school. In addition to being aware of the pitfalls of over-exhaustion (“[…] this [perfectionism in lesson-planning] is at ticket to burnout and I want to avoid it”; NQT8, all translations by third author), the participants experience a lack of work-life balance and are deliberately focusing on it:

“I want to pay particular attention to my work-life balance this week. My goal is to consciously take time for learning/working hours, to take active breaks and to plan more time for myself. I don’t want to sit at the desk from morning to night and this about every day” (NQT4).

Beyond that NQT expressed added strain when being in contact with colleagues experiencing emotional exhaustion.

Regarding the challenges experienced by the study participants, several themes seemed especially important to participants: online teaching and COVID-19, future-related matters, teacher–student relationships, as well as teacher–parent relationships. Given the global pandemic situation, the support group participants were involved in unpredictable circumstances at school. For example, one NQT noted as: “Constantly changing rules that bring a lot of ambiguity in school and also break the routines are extremely frustrating and tiring. For the children as well as for us” (NQT5). In the same passage, the participant expressed the hope to find “a good way of dealing with it” (*ibid*.) through the discussions in the peer support group. Related to this aspect was the topic of online teaching and its associated challenges, especially not “loosing” children who are, for instance, quarantined (*cf*. NQT33). Furthermore, NQTs were confronted with worries, some of them quite general regarding organization, teaching, and dealing with problems and new situations. Other worries regarded the prospects of teachers, like finding an apprenticeship. Another challenging aspect, mentioned by NQTs, was related to relationships with students’ parents. By advocating for their children, parents are prone to deny cooperation with teachers, in some cases going to the extreme of personal threats (*cf*. NQT2) and accusations (*cf*. NQT3).

In contrast to the before mentioned challenges, there were more specific themes, including challenges with individual pupils or classrooms, such as harassment among pupils and (intentional) disruptive behavior of individuals, which affect the teaching process. One study participant illustrates one pupil as having language barriers, which creates a challenge of adequate inclusion and treatment. Questions arising in this situation regard handling not only language barriers, but also tackling the problem of bullying (*cf*. NQT3). The workbook’s content further provided information about the NQTs’ handling of the challenges and problems they face. NQTs mentioned a variety of coping mechanisms such as looking for answers and solutions in literature, seeking discussion with other colleagues, and sharing their experiences on managing stressful situations with one another.

Another aspect affecting NQTs’ well-being is teachers’ attitude toward school and teaching. It can be concluded that NQTs are highly engaged in their teaching and want to offer pupils a safe space and beneficial conditions for learning and individual development. These efforts are illustrated by the following utterance:

“For me, the students are still the focus of my teaching, and I would also describe my teaching activity as student-friendly. It is important to consider the personal aspects and pathos of the individual student and not only to see them as objects to be taught” (NQT22).

Moreover, some NQTs emphasized on the importance of satisfaction, that is, the enjoyment needed in their job. It seems to be of great importance to be aware of one’s own effort and that the job is done in the best possible way. This points to another aspect of the well-being construct: the academic self-concept, that is, what teachers conceptualize as being a professional or, in other words, “a good teacher.” While there are few remarks on the kind of teachers the participants want to be(come), there are representations of handling different circumstances, which implicitly disclose deliberations that indicate a professional attitude toward teaching-related matters.

#### Social Support

As far as social support is concerned, the analysis reveals following: The NQTs emphasize commonalities such as similar pedagogical approaches (NQT19) and experiences important with regard to being able to understand what each one is going through. The group meetings primarily consist of exchange of experiences, feedback, tips, coping strategies, as well as the development of solutions for problems and planning of viable actions. It seems, however, that the commonalities are an underlying condition for the exchange to be perceived as supportive. Thereby, the NQT observe a contribution to their overall well-being:

“We give each other advice; often one person has experienced a similar situation. I also noticed that sometimes we have very similar topics or that we are all the same with stress, a lot of work, etc. It’s good to know that others are like that too!” (NQT4)

The overall peer support group climate also speaks of a supportive environment and is described by the NQTs in the words “supportive,” “appreciative,” and “relaxed.” One participant’s review on the third meeting concluded as:

“At the moment, all colleagues are treated in a relaxed and appreciative manner. Making things up, helping each other, laughing together and talking about personal things – all this has worked out great lately” (NQT13).

Indeed, most of the NQTs perceived one another as sincerely engaged in the meetings and the topics discussed. They experienced a respectful exchange in, what they called, a “good manner,” that is, everyone was willing and able to contribute and share their thoughts, experiences, and worries.

#### About the Design

Regarding the design, the NQTs reflected about the conditions for a successful peer support group. First, they identified the need for coordination between the participants. Captured in one’s daily routine, which comes with a lot of work and time constraints, there is a great need of coordination regarding meeting times. Second, both, adequate equipment (internet connection) as well as an appropriate environment (quiet room, with the possibility of little distraction) are important. One participant reported of participating in the meeting from school, which turned into meeting technical difficulties on the school’s computer and being interrupted by pupils, which lead to the realization of premeditation and better organization (*cf*. NQT31). Third, a “safe space” for exchange needs to be guaranteed. NQTs need to be able to feel like they can open up with and trust one another in order to have fruitful, helpful discussions. This is particularly highlighted by two participants struggling with it, due to reasons of confidentiality (*cf*. NQT14) and lack of familiarity with one another (*cf*. NQT3).

Alongside the above-mentioned conditions, the NQTs noted a few points that could further improve the underlying design. Those recommendations included enhanced guidelines for individual as well group-wise preparation for the sessions. As a “group rule” every group member should prepare the topics of discussion in advance and the group should at least formulate its intentions for the next meeting. Furthermore, every meeting should follow clear goals, which should drive the discussions. Some groups had repeatedly experienced deviations from the actual goal they set for the specific meeting. While some concluded that they were fine with the open approach (*cf*. NQT9), others might have viewed this as counterproductive and insisted on the importance of re-focusing (*cf*. NQT2). Finally, rules for communications, like when and how much the participants should talk, as well as a “rule for confidentiality” (NQT3) should be implemented.

Choosing a moderator for each meeting was considered an important task at the beginning of each session. It was mentioned that defining roles for each meeting may contribute to a more structured and thereby fruitful discussion with every person having a fair time to facilitate the discussion.

Two other points concerned the temporal aspect. While NQTs emphasize the need of taking more time for the sessions and the need of having a flexible time frame to not experience time pressure, one participant expressed the need of setting a clear time frame for the meetings to constrain the conversation and make it more productive (*cf*. NQT20). Lastly, specifically concerning the online format, the NQTs underline the importance of being able to see the other peer group members, even if it is only through technology. Being able to interact with the other peer support group participants in a video-based format improved not only the communication but the perception of this design as a whole.

In conclusion, the following was hold in prospect regarding the peer support group: “This system was new to me, but I firmly believe that everyone can benefit from it. Hopefully, it will be implemented in other seminars, as I have learned more through it than in most pedagogical seminars put together” (NQT7).

## Discussion

The present study explored well-being of NQTs and the potential benefits of peer support for NQTs’ well-being in a digital setting, using a sample of NQTs in Austria. Previous studies linked peer social support to well-being among NQTs. Our data complement this literature by focusing on a narrow domain: We evaluated one specific design in the specific context of the COVID-19 pandemic. Therefore, the present research contributed to research on social support and well-being in two major ways. First, we focused on a particularly critical and vulnerable time in the teaching profession and studied the sample of teachers in the beginning of their career. It has been repeatedly reported that many novice teachers leave the profession during the first years (e.g., Achinstein, 2006; Fantilli and McDougall, 2009; Hentges, 2012; Smith and Ulvik, 2017); the primary reasons for leaving have been found to be the starting teaching experience, working conditions, and the lack of support (Achinstein, 2006; Rots et al., 2007). While the COVID-19 pandemic did exacerbate the solution directly by adding further stress on the NQTs, it also affected well-being in an indirect way by blocking one known alleviation—social support. Recent studies have shown that in the on-going pandemic not the quantity of social interactions, but their quality is important for an individual’s well-being (Prinzing et al., 2021; Van Tilburg et al., 2021, p. 202). Based on this premise, the designed intervention provides a space in a digital environment for developing quality relationships with other novice teachers and allows to monitor the changes in participants’ well-being. We assessed the utility of a fully digital peer support group to investigate whether this still is a viable option in an era of physical distancing.

Second, we devised a theory-driven peer support group intervention to provide NQTs the opportunity to engage in providing and receiving emotional and social support and to explore the potential benefits of peer support on NQTs’ well-being. Next to the research-related outcomes and contributions of this study, it is important that it also delivers an evidence-based, low barriers template for a practical intervention into teacher’s well-being.

The quantitative results showed promising effects for most dimensions of well-being. Despite the relatively low sample sizes (that are, however, quite common to design-based research approaches), even statistical significance was achieved for five out of six dimensions of well-being. This points toward the usefulness of the designed social support group to improve NQT’s well-being. The areas of improvement are also echoed by the qualitative findings—the categories that were discussed in the workbooks match with the dimensions present in our theoretical model of teacher well-being. These findings are in line with the theoretical frame presented above (e.g., Bal et al., 2003; Chu et al., 2010; Rueger et al., 2016; Van Tilburg et al., 2021).

The one dimension where we did not find a statistically significant effect is positive attitudes toward school, as the NQTs did not score higher in this dimension comparing the pre-test, the post-test, and the measurement in between. This can be explained because while attitudes can sometimes change swiftly, they are resistant to change at other times; especially strong attitudes, those that are important to us, may not change very quickly (Rydell et al., 2006). In so far, it seems unsurprising that the effect could not be captured in the relatively short time frame of this study.

Both the quantitative findings and qualitative results about social support implied that the design is useful for NQT’s well-being. While this is an important finding on its own, it needs to be complemented with further research to investigate in how far it is useful *enough* to retain NQTs in schools. For example, this may include more longitudinal research that directly captures retention and intentions to leave as main concepts—this may also be a useful approach to investigate direct effects on the positive attitude toward school, for which our study setup was arguably too short. This especially also includes a closer study of the comparison group; in our particular, study setup the return of data for the comparison group was too little to perform longitudinal analyses. Also, further variations and iterations of the design may be tested for practical improvement purposes. For instance, this may include social support groups also including coaches or more senior teachers (see, e.g., Hasbrouck, 1997), although previous research does indicate that peers are preferred by the NQTs themselves (Colognesi et al., 2020). As outlined in the results, a qualitative theme emerged that participants, too, valued the connection that was created among equals. That said, trust achieved *via* “closure” is only one of the two major pathways to social capital creation as discussed by Burt (2001, 2005), the other one, brokering across (formal) group’s boundaries, may bring a different set of benefits (but, of course, also challenges). In addition, further research may investigate how individuals’ attribute moderates the effectiveness of the design (previous research has identified different teacher profile when it comes to autonomy and collaboration, which could be relevant here, too; Vangrieken et al., 2017; Vangrieken and Kyndt, 2020).

The social processes in the support group were evaluated by the NQTs to be functioning extremely well. This was echoed also in the qualitative analyses. Still, we consider it a meaningful pathway for the future to collect data that is more sensible to the details of this subject. A more fine-grained scale, or a relational approach such as social network analysis (Froehlich, 2021), may be useful tools that allow for more detailed implications.

It is a strength of design-based research to focus on one specific context and design. However, it also is a limitation when it comes to generalizability (which, however, was not a goal of this study). Replications in more contexts, and especially heterogeneous contexts, are needed to investigate the generalizability of the design. Also, social support was measured at a rather abstract level that just identified two stakeholders: the individual NQT and the rest of the group. While this approach is not uncommon in the field (Froehlich et al., 2017, 2021; Beausaert et al., 2021), other methods that take a more granular perspective—such as social network analysis (Froehlich and Brouwer, 2021)—may be more useful for identifying the role of individuals’ attributes (e.g., the competence to give or take social support) and individual relationships.

Last, we discuss implications for practice and for further improvement of the design. The feedback received by the NQTs through the workbook was very direct and detailed and was already presented in the results; we, therefore, refrain from repeating these points here. The majority of the points mentioned address the “norming stage” of group development (Johnson et al., 2002). NQTs could be given more guidance in the process of defining group norms and expectations of individuals, for example, by respective notes and reflective exercises in the workbook or asynchronous trainings (to maintain the low-cost nature of the design). Also, the proposition of meeting roles (such as moderators or timekeepers) may be a useful addition to give structure to the meetings. In general, these points may also be seen from the perspective of competency already hinted at above: In how far are NQTs competent in this type of setting to provide and take social support adequately or, aiming higher, in an optimal way? Further resources and training for group participants in terms of how to be a productive peer-coach and a peer-coachee may be useful (Robbins, 1991; Eriksen et al., 2020).

## Data Availability Statement

The raw data supporting the conclusions of this article will be made available by the authors, without undue reservation.

## Ethics Statement

The used scales were reviewed and approved by University of Bern as part of the WESIR project. The patients/participants provided their written informed consent to participate in this study.

## Author Contributions

DF, JM, and UH: conception and design and revised the manuscript. DF and UH: obtaining data. DF: quantitative analysis. DG: qualitative analysis. DF and JM: drafted the manuscript. All authors contributed to the article and approved the submitted version.

## Funding

Open Access publication fees were paid for by the University of Vienna.

## Conflict of Interest

The authors declare that the research was conducted in the absence of any commercial or financial relationships that could be construed as a potential conflict of interest.

## Publisher’s Note

All claims expressed in this article are solely those of the authors and do not necessarily represent those of their affiliated organizations, or those of the publisher, the editors and the reviewers. Any product that may be evaluated in this article, or claim that may be made by its manufacturer, is not guaranteed or endorsed by the publisher.
